# Impact of Obstructive Sleep Apnea On In-Hospital Outcomes in Patients With Atrial Fibrillation: A Retrospective Analysis of the National Inpatient Sample

**DOI:** 10.7759/cureus.20770

**Published:** 2021-12-28

**Authors:** Ahmed Brgdar, Jin Yi, Ahmad Awan, Mohamed Taha, Richard Ogunti, John Gharbin, Mehrotra Prafulla, Isaac Opoku

**Affiliations:** 1 Internal Medicine, Howard University Hospital, Washington, DC, USA; 2 Cardiovascular Disease, Howard University Hospital, Washington, DC, USA

**Keywords:** inpatient mortality, hospitalization, cardiovascular, atrial fibrillation, obstructive sleep apnea

## Abstract

Background

Obstructive sleep apnea (OSA) is frequently seen with atrial fibrillation (AF) and is associated with increased cardiovascular morbidity, including hypertension, congestive heart failure, ischemic heart disease, and stroke. However, the impact of OSA on in-hospital outcomes of patients with AF is unclear.

Methodology

All patients aged ≥18 admitted primarily for AF between January 2016 and December 2017 were identified in the National Inpatient Sample database. They were then categorized into those with OSA and those without OSA. The primary outcome was in-hospital mortality. Unadjusted and adjusted analysis was performed on appropriate variables of interest.

Results

Of 156,521 primary AF hospitalizations, 15% of the patients had OSA. Baseline characteristics revealed no race disparity between the two groups. However, compared to those without OSA, the OSA group was younger and had a significantly higher proportion of males, obesity, heart failure, hypertension, chronic obstructive pulmonary disease, diabetes, and hyperlipidemia. Long-term anticoagulation and inpatient cardioversion were also higher in the OSA group. Following propensity matching, inpatient mortality was similar between the two groups [0.54% in OSA vs. 0.51% in non-OSA; adjusted odds ratio = 1.06 (95% confidence interval = 0.82-1.35)]. Similarly, OSA was not significantly associated with acute kidney injury, cardiac arrest, gastrointestinal bleed, acute stroke, or length of stay. However, the OSA group was less anemic and required fewer in-hospital blood transfusions.

Conclusions

Although OSA is highly prevalent in AF patients, inpatient mortality and cardiovascular outcomes such as cardiac arrest, stroke, or major bleeding were similar in AF patients with or without concomitant OSA with no significant differences in length of stay.

## Introduction

Atrial fibrillation (AF) is the most common sustained arrhythmia globally, with a higher incidence and prevalence reported in developed countries compared to developing countries [1,2]. Currently, AF affects approximately six million patients in the United States alone and accounts for more than 454,000 hospitalizations each year [3,4]. Although a higher prevalence of AF has been reported with increasing age and among males, significant differences in AF prevalence by race and ethnicity have also been reported [5]. For example, in the Multi-Ethnic Study of Atherosclerosis (MESA) study, the age- and sex-adjusted incidence rates per 1,000 person-years of AF were 11.2 among non-Hispanic whites compared to 6.1 among Hispanics, 5.8 among non-Hispanic blacks, and 3.9 among Asians [6].

Patients with AF are disproportionately affected by obstructive sleep apnea (OSA) than patients without AF, with the prevalence of OSA in AF patients estimated between 21% and 74% [1]. In addition, OSA is an independent risk factor for several cardiovascular conditions such as coronary artery disease (CAD), myocardial infarction, systemic hypertension, pulmonary hypertension, and stroke [7-11]. Furthermore, OSA is associated with a significantly elevated risk of sudden cardiac death (SCD) [12,13] and is an independent risk factor of stroke in patients with AF [14].

In addition, OSA has been associated with incident AF. In a large cohort of 3,542 obese sleep clinic patients under the age of 65, those with OSA had a two-fold higher risk of incident AF within five years of an OSA diagnosis than those without OSA [15]. Similarly, in another sleep clinic-based study with 6,841 predominantly middle-aged obese patients, OSA diagnosis and severity were independently associated with incident AF over 12 years of follow-up [16]. Among participants of the MESA cohort who were free of cardiovascular disease at baseline, those with a physician-diagnosed OSA had a 1.74-fold higher risk of incident AF during an average 8.5-year follow-up period [17]. These associations between AF and OSA are expected because they share many common risks factors such as increased age, sedentary lifestyle, extreme physical activity, smoking, excessive alcohol intake, obesity, hypertension, diabetes, coronary heart disease, myocardial infarction, and heart failure, all of which may induce structural and electrical remodeling of the atrium [2,5,18].

Notably, the presence of OSA in AF patients is known to decrease the effectiveness of both pharmacological and catheter-based pulmonary vein isolation (PVI) anti-arrhythmic treatment strategies [1]. Consistent with these observations, a retrospective analysis of Outcomes Registry for Better Informed Treatment of Atrial Fibrillation (ORBIT-AF) showed that OSA patients treated with continuous positive airway pressure (CPAP) treatment, the ﬁrst-line therapy for OSA, were less likely to develop the permanent AF subtype compared to untreated patients [19]. Furthermore, among patients with AF and OSA, the risk of AF recurrence after electrical cardioversion or PVI was lower among CPAP users than non-users [20,21].

Although the relationship between AF and OSA has been relatively well-studied, there is a paucity of data reporting the impact of OSA on clinical outcomes in AF patients. In the one study we could identify, Holmqvist et al. reported that AF patients with OSA in the ORBIT-AF registry had a higher risk of hospitalization than those without OSA but had a similar risk of myocardial infarction, stroke, and cardiovascular-related mortality [19]. Moreover, little is known about in-hospital outcomes for patients with OSA and AF. Given these gaps in our understanding, we aimed to determine the impact of OSA on in-hospital outcomes in AF patients using data from the US National Inpatient Sample (NIS) database.

## Materials and methods

Data source

The NIS is a public inpatient healthcare database developed and maintained by the Healthcare Cost and Utilization Project (HCUP) under the sponsorship of the Agency for Healthcare Research and Quality to make national estimates of healthcare utilization, cost, quality, and outcomes [22]. NIS data are available from 1988 to 2019 with 48 participating states and the District of Columbia. Over seven million individual hospitalizations are recorded annually in the NIS database and include the principal diagnosis (primary discharge diagnosis), up to 29 secondary diagnoses, length of stay, up to 15 medical procedures performed during hospitalization, and total hospital costs.

The NIS databases adopted a self-weighing design in 2012 to represent a 20% stratified sample of all discharges from all HCUP-participating hospitals, covering nearly 97% of the US population in contrast to representing discharges from sampled hospitals before 2012 [22]. However, NIS excludes rehabilitation and long-term acute care hospitals [22]. Results from the NIS have been shown to correlate well with other hospitalization and discharge databases in the United States.

Study population

In this study, patients older than or equal to 18 years of age and hospitalized between January 2016 and December 2017 with AF as the primary diagnosis were identified from the NIS database using the International Classification of Diseases, Tenth Revision (ICD-10) diagnosis code. The ICD-10 codes I48.20 corresponding to chronic AF and I48.91 corresponding to unspecified AF were used to identify the patient sample for this study. The study sample was categorized into two groups: AF patients with OSA (ICD-10 = G47.33) and AF patients without OSA. A propensity score-matched cohort was developed between AF with OSA and AF without OSA groups using a 1:1 nearest neighbor matching with a caliper of 0.01.

Patients’ baseline characteristics included age, sex, race/ethnicity, household income, insurance type, length of hospital stay, and comorbidities. Hospital characteristics included location (urban and rural). We then compared clinical outcomes among AF patients with OSA and those without OSA.

Outcomes

The primary outcome of this study was in-hospital mortality among AF patients with or without OSA. Secondary outcomes included acute kidney injury (AKI), acute stroke, cardiac arrest, gastrointestinal (GI) bleed, the need for blood transfusion, and length of hospital stay. Demographic and comorbid factors were identified as covariates.

Statistical analysis

Data were analyzed using Software for Statistics and Data Science (STATA V.14.2, Stata Corp., College Station, TX, USA). Descriptive statistics were used to describe the characteristics of AF patients with or without OSA. Categorical variables were presented as percentages and continuous variables as median ± interquartile range. We employed the propensity score method with standardized morbidity ratio (SMR) weighting to account for potential confounders.

SMR weight was calculated for each patient. Patients who had OSA were assigned a weight of 1, while those without OSA were weighed using PS/(1-PS). SMR weights standardized the distribution of measured demographic, hospital, and hospital characteristics in OSA patients to those without OSA [23]. The balance was assessed once the weights were applied by examining the standardized mean differences (SMD) of the two groups. SMD (calculated as the differences in means or proportions divided by a pooled estimated of the SD) is not as sensitive to sample size compared with traditional significance testing, and it helps identify clinically meaningful differences. A threshold of >10% in the absolute SMD was used as a significant imbalance between the two groups.

Outcome measures were compared between the two groups using the P-values of the chi-square unpaired t-tests. All statistical tests were two-sided, and tests with P-values of <0.05 were considered significant. Propensity score matching was performed using the MatchIt package for R software (R for Windows 3.2.4; The R Foundation for Statistical Computing, Vienna, Austria). Howard University Hospital Institutional Review Board exempted this study from a full review because it was determined to be a non-human study. We have utilized anonymized data available from a public data repository.

## Results

We identified a total of 156,521 patient records for AF hospitalizations, of which 23,678 (15%) had concurrent OSA. Baseline characteristics revealed no race disparity between the two groups, as shown in Table 1. However, patients with OSA were younger (65 ± 10 vs. 71 ± 13, p ≤ 0.01; SMD = 53%) compared to those without OSA. The OSA group also had a significantly higher proportion of males (64% vs. 49%, p < 0.01; SMD = 32%), obesity (50% vs. 14%, p ≤ 0.01; SMD = 71%), heart failure (42.9% vs. 36.6%; SMD = 12.77%), hypertension (83% vs. 77%; SMD = 6.24%), chronic obstructive pulmonary disease (33% vs. 24%; SMD = 20.32%), diabetes (41% vs. 27%; SMD = 29.19%), and hyperlipidemia (58% vs. 49%; SMD = 17.60%). Long-term anticoagulation (35% vs. 29%; SMD = 4.04%) and in-patient cardiac cardioversion (27% vs. 18%; SMD = 20.87%) were also higher in the OSA group (Table 1).

**Table 1:** Baseline patient characteristics before and after matching. COPD: chronic obstructive pulmonary disease; OSA: obstructive sleep apnea; SMD (%): standardized mean difference (in percentage); STEMI: ST-segment elevation myocardial infarction

Variables	Pre-match: unmatched cohort (OSA, n = 23,678; non-OSA, n = 132,843)			Post-match: matched cohort (OSA, n = 23,678; non-OSA, n = 23,678)		
	OSA	Non-OSA	SMD (%)	OSA	Non-OSA	SMD (%)
*Age	65.184 ± 10	71.4 ± 13	52.94%	65.1847 ± 12.7	65.0865 ± 12.7	0.83%
*Female gender	36.21%	51.9%	32.67%	36.21%	36.19%	0.04%
Insurance type						
Medicare	57.65%	69.82%	24.62%	0.5765%	0.5833%	1.37%
Medicaid	6.80%	6.26%	2.14%	6.80%	6.32%	1.93%
Private	30.66%	19.36%	24.50%	30.66%	30.60%	0.12%
Other insurance	4.89%	4.56%	1.52%	4.89%	4.75%	0.63%
Race/Ethnicity						
Caucasian	80.83%	79.18%	4.21%	80.83%	82.02%	3.02%
Black	8.83%	7.98%	2.99%	8.83%	8.47%	1.25%
Other	6.80%	9.95%	12.5%	6.80%	6.26%	2.16%
Hospital teaching status						
Urban teaching	68.59%	61.67%	14.90%	68.59%	69.10%	1.10%
Comorbidities						
Hypertension	82.96%	76.85%	16.24%	82.96%	83.17%	0.57%
Obesity	50.57%	14.74%	71.72%	50.57%	49.98%	1.17%
Overweight	1.48%	1.54%	0.53%	1.48%	1.45%	0.24%
Coronary artery disease	35.28%	32.54%	5.73%	35.28%	35.16%	0.25%
Heart failure	42.92%	36.60%	12.77%	42.92%	41.58%	2.70%
COPD	33.05%	23.49%	20.32%	33.05%	31.79%	2.68%
End-stage renal disease	2.61%	2.57%	0.21%	2.61%	2.64%	0.19%
Chronic kidney disease	17.77%	15.44%	6.09%	17.77%	17.27%	1.30%
Diabetes mellitus	40.86%	26.51%	29.19%	40.86%	40.25%	1.25%
Hyperlipidemia	57.78%	49.08%	17.60%	57.78%	58.55%	1.57%
Smoking	42.13%	36.95%	10.48%	42.13%	41.98%	0.30%
Pulmonary hypertension	1.40%	0.95%	3.85%	1.40%	1.28%	1.01%
Malignancy	3.54%	5.50%	10.57%	3.54%	3.34%	1.10%
Alcohol abuse	4.42%	5.22%	3.90%	4.42%	4.18%	1.17%
Drug abuse	2.10%	2.36%	1.84%	2.10%	1.92%	1.24%
Hemorrhagic stroke	9.68%	11.06%	4.65%	9.68%	9.38%	1.04%
Cardiogenic shock	0.54%	0.51%	0.33%	0.54%	0.52%	0.29%
Other shocks	0.19%	014%	0.99%	0.19%	0.16%	0.59%
Hypotension	5.99%	6.71%	3.02%	5.99%	5.55%	1.89%
STEMI	1.82%	2.43%	4.50%	1.82%	1.73%	0.73%
Long-term anticoagulant	35.26%	28.55%	14.04%	35.26%	34.68%	1.21%
Cardioversion	26.93%	17.67%	20.87%	26.93%	26.46%%	1.07%

Following propensity matching, inpatient mortality, our primary outcome of interest, was similar between the two groups [0.54% in OSA vs. 0.51% in non-OSA, adjusted odds ratio = 1.06 (95% confidence interval (CI) 0.82-1.35)] (Table 2). There was no statistically significant difference between the two groups with regards to AKI (p = 0.38), cardiac arrest (p = 0.24), GI bleed (p = 0.563), acute stroke (p = 0.072), or length of stay (p = 0.67). However, the OSA group was less anemic and required fewer in-hospital blood transfusions (0.8% vs 1.0%, p = 0.04; 95% CI = 0.68-0.98).

**Table 2:** Association of OSA and outcomes in patients with AF. *Length of stay among those who survived till hospital discharge. aOR: adjusted odds ratio; AF: atrial fibrillation; CI: confidence interval; OSA: obstructive sleep apnea

	OSA	Non-OSA	aOR (95% CI)	P-value
Inpatient mortality (%)	0.54	0.51	1.06 (0.82-1.35)	0.65
Acute kidney injury (%)	12.3	12.5	0.98 (0.92-1.03)	0.38
Acute stroke (%)	0.35	0.40	0.75 (0.59-1.02)	0.072
Mean length of stay* (days)	3.4	3.43		0.67
Cardiac arrest (%)	0.38	0.31	1.20 (0.88-1.63)	0.24
GI bleed (%)	1	1.10	0.563 (0.79-1.13)	0.24
Need for blood transfusion (%)	0.80	1	0.83 (0.68-0.98)	0.04

## Discussion

Whether OSA is associated with higher inpatient mortality has been controversial. Some studies have shown that OSA is associated with a reduction in both intensive care unit and hospital mortality [24,25]. Furthermore, a recent nationwide analysis using the 2004-2014 NIS data showed that patients with concomitant diagnoses of AF and OSA have lower inpatient all-cause mortality than those with only AF [26]. In contrast, an analysis of the ORBIT-AF registry demonstrated worse symptoms and higher risks of hospitalization but similar mortality, major adverse cardiovascular outcomes, and AF progression rates in OSA patients with AF [19]. Our study aligned more with the notion that mortality was similar between the OSA and non-OSA groups; inpatient mortality was approximately 0.5% in both groups. Moreover, AKI, cardiac arrest, GI bleed, and acute stroke, which were the secondary outcomes of this study, were similar in AF patients with or without OSA. Although earlier studies have suggested that OSA patients have a longer length of stay after cardiac surgery [27] and are more likely to be readmitted within 30 days of discharge [27,28], we found no difference in the length of hospital stay between AF patients with OSA or without OSA.

Obesity, type 2 diabetes mellitus, hypercholesterolemia, and hypertension are well-studied risk factors of both AF and sleep apnea [29]. As expected, in our study population, nearly 50% of patients in the OSA group were obese compared to 14% in the non-OSA group. We noted that a middle-aged, overweight man is a typical OSA patient; this aligns with earlier research that OSA is seen more frequently in men than women [20]. Severity in both genders increases with increasing body mass index and age, a greater report of the symptoms, and decreased nadir saturation during sleep study [30,31]. Our study reaffirmed that both hyperlipidemia (58% vs. 49%; SMD = 17.60%) and hypertension (83% vs. 77%; SMD = 16.24%) were more common in patients with OSA admitted for AF. This was consistent with studies supporting OSA as the most prevalent secondary contributor to elevated blood pressure in patients with resistant hypertension [32]. Increased sympathetic activity, intrathoracic pressure during episodes of apnea resulting in hypertension, and excessive rates of venous return have been postulated as possible mechanisms for the development of AF in OSA [33].

Marked racial differences exist in the association of OSA with cardiovascular disease (CVD), and some studies have demonstrated a disproportional burden of CVD among blacks with a marked racial disparity in care and outcomes in the United States [34-36]. However, the baseline characteristics of our study population did not show race disparity between the OSA group admitted for AF and the non-OSA group, which was consistent with most of the available data on the prevalence and risk factors of OSA conducted among whites of European descent, US Hispanics, and African Americans [37]. Despite the lack of data in Asia, the prevalence is approximately 2.1-7.5%, comparable to the Caucasian population [38].

Long-term anticoagulation was higher in the OSA group than in the non-OSA group, consistent with patients in the ORBIT-AF registry [19]. The prophylactic use of anticoagulants is in line with findings that AF patients with OSA have higher CHADS2 and CHA2DS2-VASc scores, which predicts a higher risk of stroke [39]. Moreover, OSA is a contributing cause to the progression of paroxysmal AF to persistent AF [40], and in patients receiving anticoagulation therapy, those with persistent AF have a higher risk of stroke with worse survival compared to those with paroxysmal AF [41]. Moreover, the OSA group had higher inpatient cardioversion than AF patients without OSA. It has been hypothesized that CPAP treatment for OSA may help maintain sinus rhythm after electrical cardioversion [1], although a recent randomized trial showed no differences in AF recurrences after direct current cardioversion between those treated with CPAP versus usual care [42]. Additional studies are needed to evaluate CPAP adherence and its utility in preventing recurrence of AF.

Earlier studies have also suggested that OSA might be a risk factor for SCD. A retrospective study found that the relative risk of SCD was 2.57-fold higher between midnight and 6 a.m. in patients with OSA compared with the general population [12]. A 2013 longitudinal study showed that nocturnal hypoxemia, a critical pathophysiological feature of OSA, strongly predicted SCD independent of well-established risk factors [13]. These electrophysiologic changes associated with OSA may contribute to nocturnal SCD in patients with channelopathies and altered repolarization [43,44]. In contrast to these findings, AF patients with OSA in our study did not have increased cardiac arrest odds than the non-OSA group (p = 0.24). Future studies with an adequately large cohort with information about OSA at baseline and a sufficient longitudinal follow-up period are warranted to clarify the association between OSA and SCD.

There are some limitations of our study that may affect the generalizability of the findings. First, our analysis was limited to in-hospital outcomes and does not reflect post-discharge care and events. Second, our analysis was not stratified by severity due to the limitation of the NIS database; hence, stratifying for AF and OSA severity in estimating the outcomes of interest is not possible. In addition, details of treatment for OSA, diagnostic modalities, and comorbidities were not available in the database. Finally, given the nature of this retrospective analysis, selection bias can be expected; however, propensity score matching was performed to avoid possible bias.

## Conclusions

Although OSA is highly prevalent in AF, inpatient mortality and cardiovascular outcomes such as cardiac arrest, stroke, or major bleeding were similar in AF patients with or without concomitant OSA with no significant differences in length of stay. However, prospective trials are needed to evaluate in-hospital outcomes of AF patients based on sleep apnea severity. In addition, universal measures to determine sleep apnea severity and guide OSA therapy for patients with AF need further investigation.
